# Temporal Changes in Physiological Responses of Bay Scallop: Performance of Antioxidant Mechanism in *Argopecten irradians* in Response to Sudden Changes in Habitat Salinity

**DOI:** 10.3390/antiox10111673

**Published:** 2021-10-24

**Authors:** Jin Ah Song, Cheol Young Choi

**Affiliations:** Division of Marine BioScience, National Korea Maritime and Ocean University, Busan 49112, Korea; jinah@g.kmou.ac.kr

**Keywords:** bay scallop, *Argopecten irradians*, antioxidant mechanism, reactive oxygen species, salinity change, digestive diverticula

## Abstract

Changes to habitat salinity may induce oxidative stress in aquatic organisms. The effect of salinity on the antioxidant function of bay scallops was investigated at 55, 70, 85 and 120% of seawater salinity (SW), with 100% SW as the control. The scallops were sampled 0, 6, 12, 24, 48 and 72 h after the salinity change to measure superoxide dismutase (SOD), catalase (CAT), hydrogen peroxide (H_2_O_2_), and lipid peroxidation (LPO) levels, as well as apoptosis in the digestive diverticula and/or hemolymph. The SOD immunohistochemistry and apoptotic response were assessed at 55% and 120% SW at 12 h. Antioxidant expressions at 55% and 70% SW peaked at 24 h or 48 h, and then decreased. At 120% SW, they increased with exposure time. The H_2_O_2_ and LPO levels at each SW increased significantly with time. A comet assay also revealed that changes in salinity increased the rate of nuclear DNA damage in all the salinity groups. Thus, variations in salinity result in significant physiological responses in bay scallops. A change in habitat salinity of 15% or more produces oxidative stress that cannot be resolved by the body’s antioxidant mechanism, suggesting that excessive generation of reactive oxygen species can lead to cell death.

## 1. Introduction

Although various aquatic animals have adapted and thrived in the marine ecosystem, biodiversity and productivity are rapidly changing due to human activities [1]. Recently, there has been a rise in seawater temperature due to industrialization, which causes ice caps and glaciers to melt rapidly [2]. Due to heavy rain and increased river water flows into the sea, the salinity of seawater is rapidly changing and the marine ecosystem is being transformed [3]. During the 1960–2015 period, the mixed layer salinity freshened by more than 0.2 psu, with an averaged trend of −0.20 psu/50 years (or −0.004 psu/year) in the South China Sea [4]. The continuous change in the salinity of sea water, with the recent trends in global warming, is one of the important ecological factors that play a decisive role in the marine ecosystem, including in the distribution of marine organisms, and affects the metabolism, osmotic pressure control, and stress in marine organisms [5].

Among marine organisms, exposure to stressful environments, such as rapid salinity changes, results in the generation of excessive reactive oxygen species (ROS) in the tissues and hemolymphs of bivalve mollusks, such as scallops as well as fish. It also induces apoptosis by damaging unsaturated lipids [6,7]. As ROS are a major cause of oxidative stress, bivalves activate their antioxidant system to defend themselves against large amounts of ROS. Superoxide dismutase (SOD) and catalase (CAT), as antioxidant enzymes, are expressed by the antioxidant protective capacity of biological systems against ROS. Toxic ROS generated in the tissues are catalytically converted to hydrogen peroxide and then converted back into non-toxic water and oxygen to protect body tissues from oxidative damage [8,9]. When an organism is exposed to a harmful environment, a defense mechanism induces the generation of antioxidant enzymes to reduce the toxicity produced, but an imbalance in the formation and neutralization of ROS leads to oxidative stress [10]; the ROS generated in excess of what can be removed by the antioxidant system leads to oxidative damage, mainly in the form of lipid peroxidation (LPO), DNA damage, and apoptosis [11]. LPO is one of the biomarkers for oxidative stress and damage, and the extent of stress or damage is determined by measuring the amount of malondialdehyde (MDA) generated during LPO [12]. DNA damage and apoptosis are central protective responses to excess oxidative stress [13].

Among the different types of tissues found in marine bivalve mollusks, digestive diverticula actively exhibit antioxidant activity in the body [12], while hemolymphs play an important role in stress recovery and phagocytosis by influencing physiological processes [14].

Scallops are widely known around the world as a target species for aquaculture and as a species with very high economic value [6]. In particular, the bay scallop is a shellfish species that is considered commercially important not only in the Atlantic coast but also in Korea and China [15]. In general, bay scallops live in shallow waters, often in environments with large inflows of fresh water, resulting in rapid variations in salinity. Nevertheless, studies on the stress responses of bay scallops to salinity changes have been limited in scope; some studies have been conducted but they focused mainly on physical changes such as growth, as well as on survival and filtration rates.

Therefore, in this study, changes in oxidative stress and apoptosis in bay scallops exposed to an environment of rapid salinity changes were investigated to establish the salinity-stress response of bay scallops. The study observed changes in SOD levels—one of the antioxidant enzymes—as well as in its mRNA and protein expression and activity to assess the antioxidant response in the digestive diverticula—a major detoxifying tissue—of bay scallops under five different salinity environments including the control. In addition, changes in CAT mRNA expression and activity, as well as changes in H_2_O_2_ and LPO levels were investigated. To confirm the degree of apoptosis due to DNA damage, a comet assay was also performed on the digestive diverticula tissue cells.

## 2. Materials and Methods

### 2.1. Experimental Species and Conditions

Bay scallops *Argopecten irradians* (average shell length of 55.0 ± 2.1 mm) were obtained from a commercial market in Goseong, Korea. Before the start of the experiment, individual bay scallops were acclimated for a week in five 400 L circulation filter tanks containing filtered and aerated seawater (temperature at 17 ± 2 °C, salinity 35‰). Half the volume of the seawater in the tank was changed daily. The scallops were allowed to acclimate to the experimental conditions for one week. 

To explore the effects of relative hypo and hyper-osmotic stress on *A. irradians*, control salinity (35‰ (salinity of seawater [SW], considered as 100%)), low salinity (19.25‰ (55% SW), 24.5‰ (70% SW), and 29.8‰ (85% SW)), and high salinity (42‰ (120% SW)) solutions were used, which were prepared from local tap water by adding Instant Ocean synthetic sea salt (Aquarium Systems, Blacksburg, VA, USA) in accurate amounts corresponding to each salinity. In the salinity acclimation experiment, *A. irradians* were suddenly transferred from control conditions to water with salinities of 55, 70, 85 and 120% SW for 72 h. During the experiment, all conditions, apart from salinity, were maintained as in the control but with no feeding. During the exposure, no mortality was observed. Five scallops from each replicate treatment group were randomly sampled at 0, 6, 12, 24, 48 and 72 h after exposure to osmotic stress. The digestive diverticula tissues were collected from each scallop and stored at −80 °C for total RNA extraction. 

### 2.2. Western Blot Analysis

The total protein content of the *A. irradians* digestive diverticula was extracted using a T-PER^®^ tissue protein extraction reagent (Thermo Fisher Scientific Inc., Waltham, MA, USA) following the manufacturer’s instructions. Protein extracts were loaded into Mini-PROTEAN^®^ TGX™ gels (Bio-Rad, Hercules, CA, USA) and transferred to a 0.2 µm polyvinylidene difluoride membrane (Bio-Rad). Membranes were incubated with SOD antibody (1:2000; TA326384, OriGene, Rockville, MD, USA) and subsequently incubated with horseradish-peroxidase-conjugated anti-mouse IgG secondary antibodies (1:4000; Bio-Rad) for 60 min. β-tubulin (1:4000, ab6046, Abcam, Cambridge, UK) was used as the internal control. The membrane images were scanned using a high-resolution scanner (Bio-Rad).

### 2.3. RNA Extraction and cDNA Synthesis

Total RNA was extracted from 30 mg of digestive diverticula tissue using the TRI reagent^®^ (Molecular Research Center, Cincinnati, OH, USA) according to the manufacturer’s instructions. Total RNA (2 μg) was then reverse-transcribed in a reaction with a total volume of 20 μL using an oligo-d(T)_15_ anchor and M-MLV reverse transcriptase (Promega, Madison, WI, USA) as per the manufacturer’s protocol. The resulting reverse-transcribed products were used for PCR amplification.

### 2.4. Quantitative PCR 

Quantitative PCR (qPCR) was conducted to determine the relative expression levels of SOD, CAT, and ribosomal protein S18 (RPS18) mRNA using the cDNA. RPS18 mRNA was used as the internal control. The primers used for qPCR are shown in Table 1. The transcription levels of the mRNAs were determined by a Bio-Rad CFX96™ Real-time PCR Detection System (Bio-Rad) using an iQ™ SYBR Green Supermix (Bio-Rad). PCR amplification was performed under the following conditions: 0.5 μL of cDNA, 0.26 μM of each primer, 0.2 mM of dNTP, SYBR Green, and Taq polymerase in buffer (10 mM Tris–HCl [pH 9.0], 50 mM KCl, 1.4 mM MgCl_2_, and 20 nM fluorescein), totaling to a final volume of 25 μL. The qPCR protocol was as follows: 95 °C for 5 min, followed by 40 cycles of 95 °C for 20 s each, and 55 °C for 20 s. Relative mRNA expressions were determined using the 2^−ΔΔCt^ method.

### 2.5. Analysis of Hemolymph Parameters

The SOD, CAT, and H_2_O_2_ activities in the hemolymph were measured with specific assay kits (SOD, BM-SOD200; CAT, BM-CAT400; H_2_O_2_, BO-PER500; and BIOMAX Co., Ltd., Seoul, Korea) following the manufacturer’s instructions. In the SOD assay, oxidation of xanthine was monitored by measuring absorbance at 450 nm. One unit of SOD activity (U/mL of hemolymph) was defined as the amount required to inhibit 50% of the oxidation process. One unit of CAT activity (U/mL of hemolymph) was defined as 50% H_2_O_2_ consumption at 1 min and with a pH of 7.0. 

### 2.6. Malondialdehyde Content

Malondialdehyde (MDA) content, as a marker of LPO, was determined in the hemolymph. The thiobarbituric reactive species (TBARS) assay was employed to determine the MDA content. These were measured spectrophotometrically at 532 nm according to the method described by Ohkawa et al. [16].

### 2.7. Detection of Superoxide Dismutase Expression by Immunohistochemistry Staining 

SOD expression in the digestive diverticula were detected immunocytochemically based on the methods described by Takata et al. [17], with modifications. First, the digestive diverticular were fixed in 4% paraformaldehyde, dehydrated in ethanol, and then embedded in paraffin. In brief, paraffin-embedded sections were deparaffinized in xylene, rehydrated in ethanol, and then incubated overnight at 4 °C with primary mouse anti-SOD antibodies (dilution 1:2000; TA326384, OriGene, Rockville, MD, USA), as well as with secondary antibodies afterwards (HRP-conjugated anti-mouse immunoglobulin; 1:1000). The antibody binding was visualized by applying 3,3′-diaminobenzidine as a detection system. Slides were counterstained and mounted with Canada balsam for observation under a light microscope (DM 100; Leica, Wetzlar, Germany); images were captured with a digital camera (DS-Fi1c, Nikon, Tokyo, Japan).

### 2.8. Comet Assay

Digestive diverticula cells (1 × 10^5^ cells/mL) were examined using a Comet Assay Reagent kit (Trevigen Inc., Gaithersburg, MD, USA) based on the method described by Singh et al. [18], with some modifications. Cells were immobilized in 1% agarose gels on comet slides and immersed in freshly prepared alkaline unwinding solution for 20 min in the dark. Next, slides were electrophoresed at 21 V for 30 min with alkaline electrophoresis solution and then dried for 15 min. The samples were stained with SYBR gold solution (Trevigen Inc.) for 30 min in the dark and then observed using a fluorescence microscope (excitation filter of 465–495 nm; Eclipse C*i*, Nikon, Tokyo, Japan). At least 100 cells from each slide were analyzed. For quantification of the comet assay, we analyzed the tail length (distance of DNA migration from the head) and percentage of DNA in the tail (tail intensity/total intensity) using comet assay IV image analysis software (version 4.3.2; Perceptive Instruments Ltd., Cambridge, UK).

### 2.9. Statistical Analysis

All data were analyzed using SPSS version 25.0 (IBM SPSS Inc., Armonk, NY, USA). For all the parameters analyzed, exposure to different salinity, as well as samples at different times of exposure were tested using two-way ANOVA. Levene’s test for equality of variances showed that there was homogeneity of variance between the treatments. Where significance was indicated, Tukey’s post hoc test for multiple comparisons was employed. The significance level adopted was 95% (*p* < 0.05). Values are expressed as the mean ± standard deviation (SD).

## 3. Results

### 3.1. Superoxide Dismutase Protein, mRNA Expressions, and Activities

**Figure 1:**
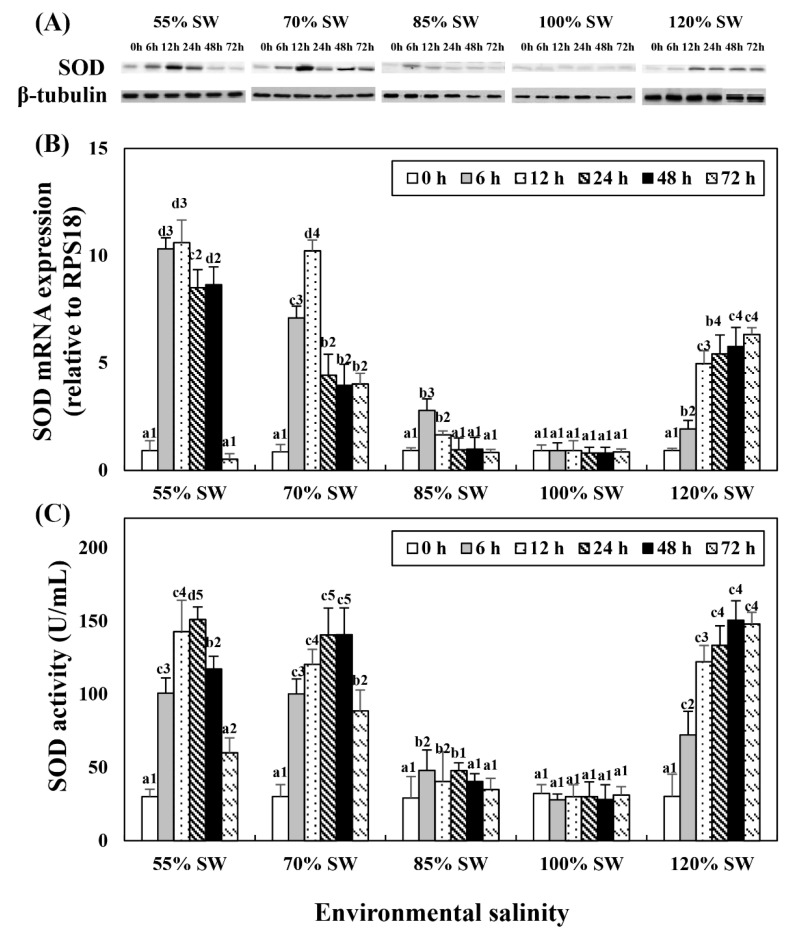
Changes in the expression levels of the (**A**) SOD protein and (**B**) SOD mRNA in the digestive diverticula, and (**C**) SOD activity in the hemolymph of *A. irradians* during a 72 h period after sudden salinity change. Different characters indicate values corresponding to different exposure times of the scallops within the same salinity concentration (*p* < 0.05). Numbers indicate significant differences among salinity concentrations for the same exposure time (*p* < 0.05). All values are presented as the mean ± SD (*n* = 5). Abbreviations: SOD, superoxide dismutase and SD, standard deviation.

Western blot analysis revealed a protein with SOD enzyme-specific immunoreactivity and mass that corresponded to the predicted mass of SOD (19 kDa; Figure 1A). This experiment was performed to compare the changes in SOD mRNA expression and activity with salinity changes during the 72 h period after salinity change (Figure 1B,C). In the 85% SW group, the SOD protein and mRNA expression levels increased at 12 h and then decreased. In the 70 and 55% SW groups, they peaked at 12 h or 24 h, and subsequently decreased. In the 120% SW group, SOD mRNA expression and activity increased with the duration of exposure. 

### 3.2. Immunochemical Staining of Superoxide Dismutase 

**Figure 2:**
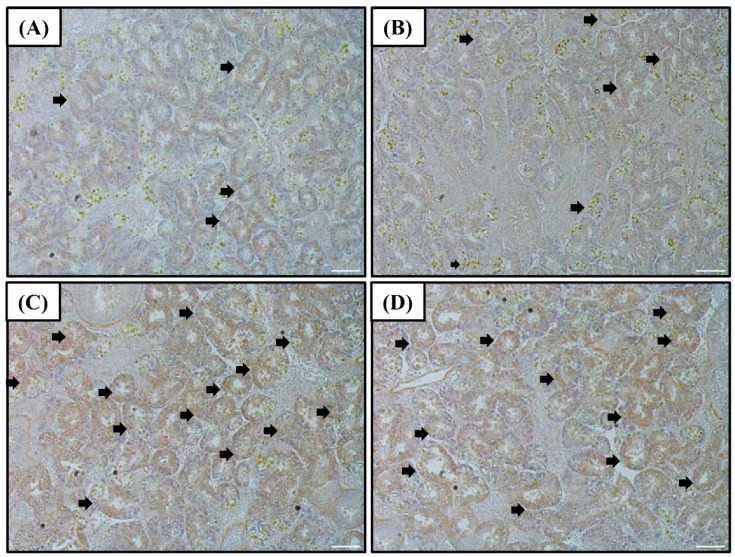
Images of the SOD immunohistochemistry of the digestive diverticula tissue of *A. irradians* after sudden salinity change for (**A**) 100% SW for 0 h; (**B**) 100% SW for 12 h; (**C**) 55% SW for 12 h; and (**D**) 120% SW for 12 h. White scale bars = 100 µm. Brown cells represent immuno-stained hemocytes and are indicated by arrows. Abbreviations: SOD, superoxide dismutase and SW, seawater salinity, 35‰.

The SOD protein levels in the digestive diverticula were found to exhibit a tendency similar to that of the SOD mRNA and protein (in western blot) expressions (Figure 2). Levels of the SOD protein in the 55 and 120% SW groups were higher than that in the control group.

### 3.3. Catalase mRNA Expression and Activity

**Figure 3:**
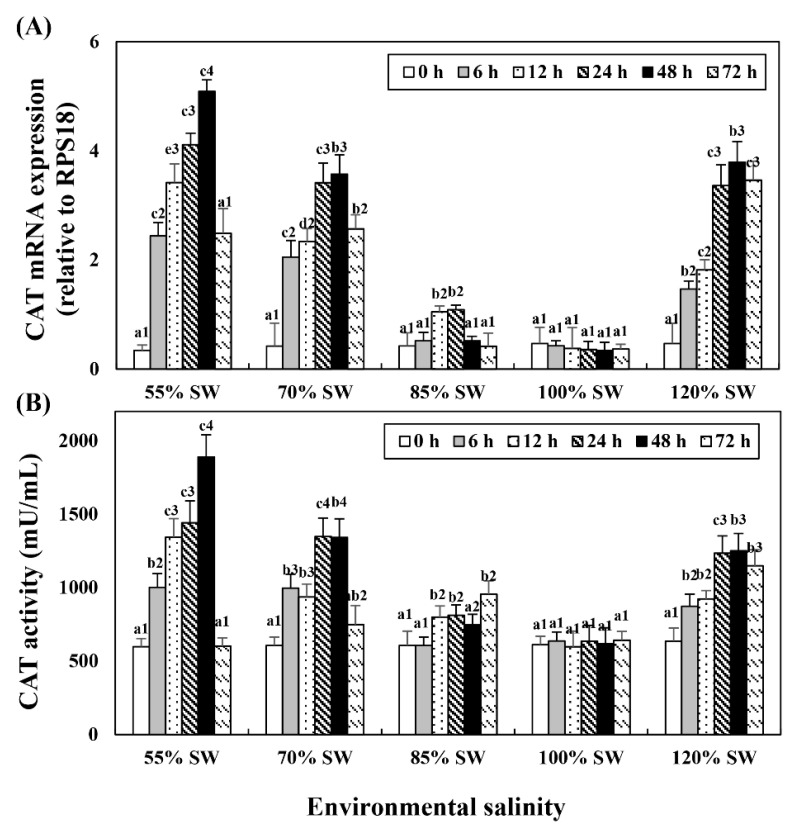
Changes in the expression levels of (**A**) CAT mRNA in the digestive diverticula and (**B**) CAT activity in the hemolymph of *A. irradians* during a 72 h period after sudden salinity change. Different characters indicate values corresponding to different exposure times of the scallops within the same salinity concentration (*p* < 0.05). Numbers indicate significant differences among salinity concentrations for the same exposure time (*p* < 0.05). All values are presented as the mean ± SD (*n* = 5). Abbreviations: CAT, catalase and SD, standard deviation.

Changes in mRNA expression in the digestive diverticula and CAT levels in the hemolymph following salinity change were investigated (Figure 3). In the 85% SW group, mRNA showed the highest expression at 24 h but subsequently decreased to the control level. However, mRNA expression and activity began to increase after 12 h and maintained the tendency to increase until 72 h. In the 70% SW group, both the expression and activity of CAT mRNA showed the highest values at 48 h and decreased to 12 h or 24 h levels at 72 h. In the 55% SW group, they peaked at 48 h and then decreased to 0 h or 12 h levels. In the 120% SW group, the HSP70 mRNA expression levels increased with the duration of exposure and the expression was significantly similar from 24 to 72 h after the salinity change.

### 3.4. Levels of Hydrogen Peroxide and Lipid Peroxidation in the Hemolymph

**Figure 4:**
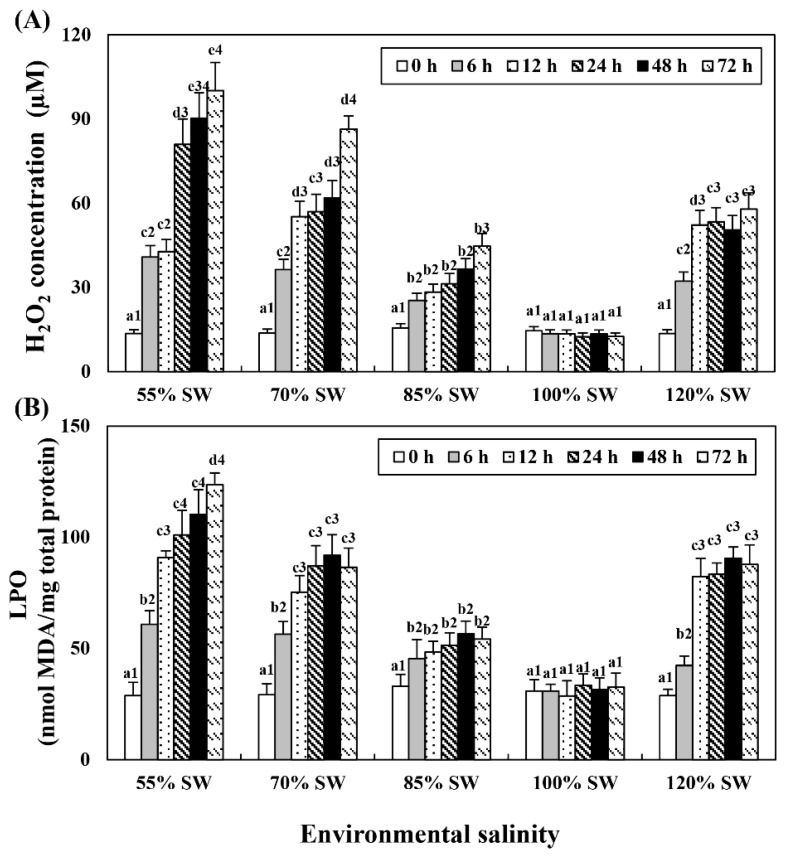
Concentration of (**A**) H_2_O_2_ and (**B**) LPO in the hemolymph of *A. irradians* during a 72 h period after sudden salinity change. Different characters indicate values corresponding to different exposure times of the scallops within the same salinity concentration (*p* < 0.05). Numbers indicate significant differences among salinity concentrations for the same exposure time (*p* < 0.05). All values are presented as the mean ± SD (*n* = 5). Abbreviations: H_2_O_2_, hydrogen peroxide; LPO, lipid peroxidation; and SD, standard deviation.

Hemolymph H_2_O_2_ and LPO levels tended to increase with the duration of exposure in all the salinity groups (Figure 4). The experimental group with the highest increase was the 55% SW group, followed by the 70, 120 and 85% SW groups.

### 3.5. Analysis of DNA Damage

**Figure 5:**
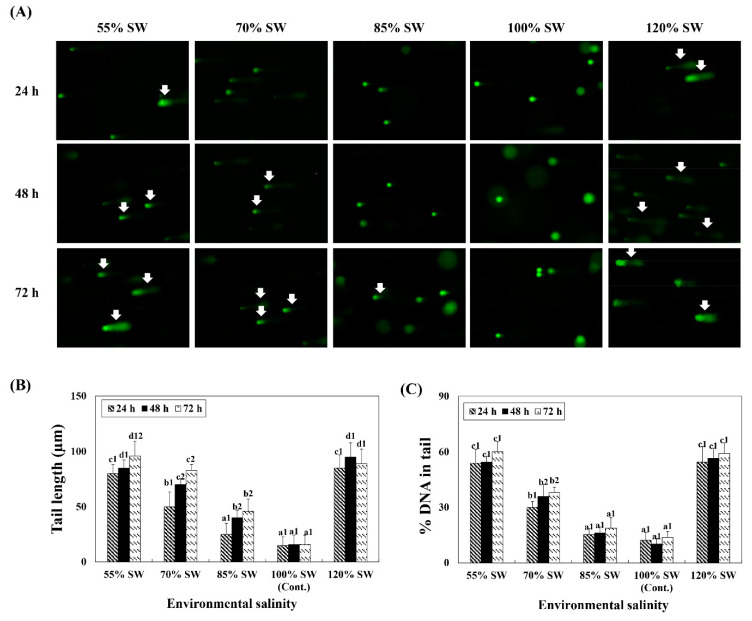
(**A**) Comet assay images and comet assay parameters, (**B**) tail length, and (**C**) DNA percentage in the tail during a 72 h period after sudden salinity change. Damaged nuclear DNA (DNA breaks) of the digestive diverticula cells were stained with SYBR-gold. White arrows indicate damaged nuclear DNA (DNA breaks) in the cells. Different characters indicate values corresponding to different exposure times of the scallops within the same salinity concentration (*p* < 0.05). Numbers indicate significant differences among salinity concentrations within the same exposure time (*p* < 0.05). All values represent the mean ± SD (*n* = 5). Abbreviation: SD, standard deviation.

DNA damage in the digestive diverticula tissue following exposure to different salinities (55, 70, 85, 100 and 120% SW) was analyzed using 100 randomly selected cells. Both the tail length and DNA content in the tail significantly increased with variations in salinity (Figure 5). In comparing DNA damage of the same exposure durations for the different salinity groups, it was observed that significantly more damages to the DNA were induced in the 55% and 120% SW groups.

## 4. Discussion

Among the various environmental factors influencing marine life, salinity is a major factor that affects the physiological responses of organisms and induces oxidative stress in them [19].

Oxidative stress occurs when ROS is excessively generated in the body by external factors. In aquatic organisms, the reaction to oxidative stress is greatly influenced by water salinity. It was reported that when *Ruditapes philippinarum* was exposed to high salinity (35 ppt) and low salinity (14 ppt) environments, the expressions of SOD and CAT increased, along with an increase in LPO levels [2]. In this study, in the 120% SW (high salinity) environment, as the period of exposure to the environment with altered salinity increased, SOD mRNA expression and activity, as well as protein expression, increased in the bay scallop. However, individuals exposed to 55% and 70% SW (low salinity) environments showed an increase in these values only for 12 h, after which they experienced a sharp decrease in these values. These results were observed to be generally similar across the groups in this study, with only a slight difference at the 48 h time point, at which CAT mRNA expression and activity started to decrease; that is, up to 12 h after exposure to an environment of different salinity, a defense mechanism generated in the body to suppress ROS was active, with the expression levels of antioxidant enzymes increasing over time. However, from that point on, it was observed that the expressions of antioxidant enzymes drastically decreased because the scallop had exhausted the body’s ability to defend itself.

Carregosa et al. [20] showed that when various clam species were exposed to different salinity environments of 0–42 ppt for 96 h, the activity of antioxidant enzymes such as SOD, CAT, and glutathione (GSH) increased at 14 ppt, which is a low-salinity environment, whereas their activity was inhibited at 35 ppt. The difference between these results and the results of our study appears to be due to differences in salinity adaptation and defense mechanisms between species.

Additionally, in this study, the expression of the SOD protein was observed through western blot and IHC to specifically confirm the expression of antioxidant enzymes in bay scallops following rapid salinity changes. First, we performed western blot on the digestive diverticula, a tissue responsible for detoxification in scallops (Figure 1A); SOD IHC was performed for the 55% and 120% SW experimental groups at 12 h, which showed almost the same high level of protein expression in all salinity groups (Figure 2). Both protein and mRNA expressions exhibited similar tendencies with regard to SOD and it was confirmed through IHC that the expression of the SOD protein significantly increased based on the extent of salinity change in the digestive diverticula cells.

These results confirmed that oxidative stress in the digestive diverticula of bay scallops increases with the changes in salinity and that an antioxidant reaction occurs to actively suppress the accumulation of the ROS generated in the body.

However, if the removal of the ROS generated by salinity stress is not adequate to resolve the toxicity induced and is accumulated in the body despite the increase in antioxidant enzymes, the LPO level increases. Therefore, in this study, the level of LPO and the degree of apoptosis were investigated to determine the degree of oxidative damage and ROS generation, which may have increased in spite of the antioxidant action in scallops exposed to an environment of rapid salinity changes. It was observed that ROS and LPO levels increased with increasing/decreasing salinity and duration of exposure. In addition, in the 12 h period when the expression of antioxidant enzymes was highest, it was confirmed through the results of the comet assay that nuclear DNA damage increased as the salinity was altered.

In noble scallop, specifically *Chlamys nobilis*, a significantly higher LPO concentration was observed in diseased scallops compared to healthy scallops [21]. Ni et al. [22] also found that when *Cyclina sinensis*—a species of bivalve mollusk—was exposed to a low salinity environment of 8 to 25‰, the expression of the inhibitor of the apoptosis protein (IAP) decreased as the salinity decreased. In addition, it was reported that in *Crassostrea virginica* exposed to high salinity and high temperature, lymphocyte apoptosis was increased [23,24].

The results of this study are consistent with those of previous studies which reported that apoptosis occurred on a large scale due to a substantial increase in ROS and LPO levels in bay scallops as the salinity of the habitat changed. This seems to indicate that greater salinity change leads to greater oxidative stress that occurs in bay scallops exposed to salinity changes. It is deduced that the problem of excessive ROS is not resolved by the increased antioxidant enzymes generated to control such oxidative stress; rather, it is generated in large amounts, leading to an increased accumulation of LPO in the body. This suggests that there is a possibility that this accumulation of LPO may ultimately lead not only to the deterioration of cell function but also to apoptosis.

This study showed that when exposed to an environment of rapidly increasing or decreasing salinity, mRNA and protein expressions, as well as the activity of antioxidant enzymes increased with changes in salinity but decreased sharply 24 h after the change occurred. This could suggest the possibility that the body’s antioxidant defense mechanism may have been restored to its normal state. However, considering that the ROS and LPO content increased despite the decrease in the expression of antioxidant enzymes, the decrease in the expression of antioxidant enzymes cannot be considered to be due to the recovery of the body’s defense mechanism but rather due to an increase in ROS—which was not resolved by the increase in antioxidant enzymes—above a certain level. It is inferred that the expression of antioxidant enzymes was suppressed by the high level of toxicity which the body’s defense mechanism was not able to resolve. This condition appears to have led to the death of digestive diverticula cells; when exposed to salinity stress for a period longer than 72 h, which was the duration of this experiment, the bay scallop could not adapt to the salinity change and subsequently cell death was observed.

## 5. Conclusions

The results, thus, provide an opportunity to confirm that salinity is a factor that has a great influence on the physiological response of bay scallops to stress. In addition, these results show that if a change in salinity of 15% (about 5‰) or more occurs in the environment inhabited by bay scallops, the oxidative stress induced cannot be resolved by the antioxidant mechanism activated in the organism and excessive generation of ROS in the cells could lead to cell death. The results of this study can be used as basic data on the effect of low habitat-salinity on bay scallops, which can occur frequently in the water masses inhabited by bay scallops in the face of increasing climate change.

## Figures and Tables

**Table 1:** Primers used for qPCR and in situ hybridization.

Genes	Primer	DNA Sequences	PCR Efficiency (%)
SOD (EU137676)	Forward	5′-TAG GGA TTT TGG CTC GTT TG-3′	104.7
	Reverse	5′-GTA GGC ATG CTC CCA AAC AT-3′	
CAT (GQ265925)	Forward	5′-TAC TGC AAG GCC AAG CTT TT-3′	111.4
	Reverse	5′-GGA ATT CCG GTC AGT TCA AA-3′	
RPS18 (AF526232)	Forward	5′-GTC TGC AAG AAG GCT GAT GT-3′	101.1
	Reverse	5′-GGG TTG GAC ATG ATT GTG AT-3′	

## Data Availability

The data presented in this study are available in the article.
